# Modulation of the human fecal metabolome – Effect of polyphenols depends on the BMI

**DOI:** 10.1016/j.crfs.2025.101060

**Published:** 2025-04-27

**Authors:** Julia Jensen-Kroll, Tobias Demetrowitsch, Sabrina Sprotte, Fynn Brix, Alexia Beckmann, Kristina Schlicht, Matthias Laudes, Mario Hasler, Charles M.A.P. Franz, Karin Schwarz

**Affiliations:** a Institute of Human Nutrition and Food Science, Division of Food Technology, Kiel University, Kiel, Germany; b Max Rubner-Institut, Federal Research Institute of Nutrition and Food, Department of Microbiology and Biotechnology, Kiel, Germany; c Division of Endocrinology, Diabetes and Clinical Nutrition, Department of Internal Medicine I, University Medical Center Schleswig-Holstein, Kiel, Germany; d Institute of Diabetes and Clinical Metabolic Research, Kiel University, Kiel, Germany; e Applied Statistics, Faculty of Agricultural and Nutritional Sciences, Kiel University, Kiel, Germany

**Keywords:** Plant compounds, Microbiome, Mass spectrometry, FT-ICR-MS, Metabolomics

## Abstract

Health effects associated with microbial metabolites are influenced by dietary compounds and other environmental factors. Polyphenols derived from plant-based foods reach the large intestine mostly undigested, where they can interact with the gut microbiota. This explorative study investigated the metabolic responses of gut microbiota to the polyphenols rutin and genistein. Ex vivo anaerobic incubations with pooled fecal samples from volunteers with a BMI <25 (n = 7) and a BMI >40 (n = 7) were analyzed by ESI DI-FT-ICR-MS. Differences in metabolic diversity were observed between the two BMI groups, with the obese group showing a less diverse metabolic response.

Metabolomic profiling identified 361 metabolites in 35 substance classes, with notable effects of the polyphenols on amino acid, carbohydrate, nucleotide, and lipid metabolism. Both BMI groups showed increased levels of dipeptides and amino acids and decreased levels of biogenic amines. Among the key findings, glutamine levels increased, which has been associated with obesity-related metabolic processes, while tryptophan levels were also elevated, a factor previously associated with obesity-related pathways. Glycine levels increased in both groups. Additionally, histamine, cadaverine, putrescine, and trimethylamine were reduced after exposure to the polyphenols.

Changes in metabolites related to carbohydrate metabolism suggest an influence of rutin and genistein on sugar transport and cell wall synthesis. Furthermore, in the obese group, rutin exposure was associated with increased butyrate levels and decreased lactate levels. These findings contribute to a better understanding of how rutin and genistein interact with the gut microbiota metabolome, with potential implications for metabolic health and obesity-related research.

## Highlights

• More diverse response to the polyphenols in BMI <25 vs. BMI >40.
• Main effects on dipeptides, amino acids, and corresponding pathways.
• Polyphenols can have both beneficial and detrimental effects on health.
• Polyphenols reduce histamine levels in BMI >40 pools.

## Graphical abstract

**Figure:**
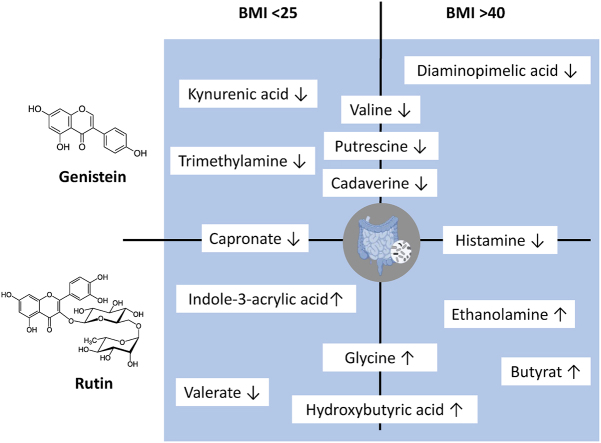

## 1 Introduction

Obesity is a complex, multifactorial disease characterized by excessive body fat accumulation, and its prevalence is rapidly rising in developed countries. This increase represents a major public health concern, as obesity is associated with numerous health problems, including type 2 diabetes, cardiovascular diseases, hypertension, inflammatory disorders, and cancer (Hruby and Hu, 2015). Several studies have revealed that the gut microbiota plays an important role in obesity, with obese individuals often having a less diverse microbiome compared to non-obese individuals (Ley et al., 2006; Turnbaugh et al., 2006, 2009). Pinart et al. (2021) identified 15 bacterial genera whose relative abundance is altered in obesity, and it is well established that the gut microbiome influences energy harvesting, fat storage, inflammation, appetite regulation, and insulin sensitivity in the context of obesity (Bäckhed et al., 2004; Musso et al., 2010; Tilg and Kaser, 2011; Turnbaugh et al., 2006).

Diet is a key factor influencing the composition and function of the gut microbiota (Scott et al., 2013; Singh et al., 2017), with plant-based compounds, particularly polyphenols, showing beneficial effects. Polyphenols, which are mostly undigested in the small intestine and reach the colon, are naturally present in fruits, vegetables, grains, and beverages such as tea, coffee, and juice (Cardona et al., 2013; Manach et al., 2004). These compounds are known for their anti-inflammatory, antioxidant, immunomodulatory, anticancer, and microbiome-modulating properties (Bié et al., 2023). Recently, the impact of polyphenols on obesity has received increased attention. These compounds help combat obesity by preventing fat accumulation, modulating inflammation by inhibiting TNF-alpha-induced NF-kB pathways, and reducing energy efficiency by inhibiting digestive enzymes (Corrêa et al., 2019). They also promote fat oxidation, thermogenesis, and lipid excretion, in addition to influencing appetite regulation (Corrêa et al., 2019; van Hul and Cani, 2019; Liu et al., 2024). Rutin and genistein are two polyphenols that have been shown to have anti-obesity effects (Hwang et al., 2005; Yuan et al., 2017).

Rutin, a glycoside of quercetin, is found in a variety of plants, including buckwheat, citrus fruits, berries, and tea. Beyond its anti-obesity effects, rutin is recognized for its anti-inflammatory, antioxidant, neuroprotective, nephroprotective, and hepatoprotective properties (Ganeshpurkar and Saluja, 2017). Similarly, genistein, an isoflavone found primarily in soy products, has antioxidant and anti-inflammatory properties. It also shows potential in cancer prevention, osteoporosis protection, cardiovascular disease reduction, and relief of postmenopausal symptoms (Messina and Messina, 2010; Rasheed et al., 2022; Sharifi-Rad et al., 2021; Goh et al., 2022).

While the effects of polyphenols are well established, their underlying mechanisms, particularly in relation to the gut microbiota and its metabolites, are only partially understood. Previous research has mainly focused on the microbial degradation products of polyphenols rather than the broader changes in microbial communities and their effects on host metabolism. A study by Ulaszewska et al. (2020) highlighted the interaction between polyphenols and the microbiome-host axis, showing that daily apple consumption affected microbial pathways related to tyrosine and tryptophan. Other studies, like Iglesias-Carres et al. (2022), showed that polyphenols can inhibit the production of trimethylamine (TMA), a compound associated with atherosclerosis. The host-microbiome axis, as proposed by Nicholson et al. (2012), highlights how the composition of the microbiomes influences the host metabolome. Studies by Henneke et al. (2022) and Wilmanski et al. (2019) have provided further support by demonstrating correlations between gut bacterial diversity, abundance, and metabolic profiles.

In our previous work (Jensen-Kroll et al., 2025), we examined how rutin and genistein are metabolized by the gut microbiota in individuals belonging to different BMI categories (BMI >40 and BMI <25). We identified 46 metabolites of rutin and 23 metabolites of genistein, some of which have not been previously described. Furthermore, the microbial community in individuals with BMI >40 showed a lower bacterial diversity compared to those with BMI <25. Polyphenol treatments affected the composition of 34 bacterial families and 83 genera, indicating a shift in microbial profiles due to these plant compounds.

This study aimed to further explore how rutin and genistein affect the microbiome-host axis by focusing on bacterial metabolic pathways and metabolites altered by these polyphenols. Using a semi-targeted metabolomics approach, we aimed to uncover how these shifts in microbial communities and their resulting metabolomic profiles might lead to postbiotic effects and whether these responses differ based on the polyphenol type and BMI category.

## 2 Material and methods

### 2.1 Sample material and sample collection

Human fecal samples were collected from two distinct groups of volunteer participants: one group (n = 7) with a BMI >40 kg/m^2^ and another group (n = 7) with a BMI between 18.5 and 25 kg/m^2^. After collection, the fecal samples were immediately transferred to sample containers and stored under cooled, anaerobic conditions in an anaerobic container with an AnaeroGen™ pack. Samples were then transferred to an anaerobic workstation and aliquoted under anaerobic conditions within 24 h according to Jensen-Kroll et al. (2025). Feces were frozen and stored at −80 °C until cultivation.

### 2.2 Ethical aspects

The use of the sample material was authorized under the ethics application AZ D 420/20 (Ethics Committee, Medical Faculty, Kiel University). Prior to the study, the volunteers were informed of the intended use of the sample material by the supervising clinician. No personal data were collected, with the only information recorded pertaining to the BMI status.

### 2.3 Anaerobic incubation experiments

Rutin and genistein had a purity of ≥98 % and were purchased from Cayman Chemical (Tallinn, Estonia). All fecal samples were transferred to the anaerobic workstation (Whitley A45, Meintrup DWS Laborgeräte, Herzlake, Germany) with an atmosphere consisting of 10 % H_2_, 10 % CO_2_, and 80 % N_2_. The incubation experiments were performed for 48 h at 37 °C and were handled as previously described by Jensen-Kroll et al. (2025). All experiments were conducted in three experimental replicates, each grown independently.

### 2.4 Sample preparation for metabolomics analyses

For the extraction, 300 μl of material was used from each sample of the cultivation experiments. Hydrophilic and lipophilic metabolites were extracted with an adapted SIMPLEX protocol and handled according to Jensen-Kroll et al. (2022). The samples were stored at −80 °C until analysis. Each experimental replicate was extracted in technical triplicates.

### 2.5 DI-FT-ICR-MS measurements

A direct injection FT-ICR-MS (7 T, SolariXR, Bruker, Bremen, Germany), equipped with an electro spray ionization source linked to a 1260 Infinity HPLC (Agilent, Waldbronn, Germany), was used. Samples were measured in positive and negative mode and in two different methods with a total mass range of 75–3000 m/z. All methods were calibrated with sodium trifluoroacetate. For further details, see Jensen-Kroll et al. (2025).

### 2.6 Data handling and metabolite annotation

FT-ICR-MS data were analyzed using MetaboScape 2021b (Bruker, Bremen, Germany). All datasets were recalibrated using commonly occurring compounds in the sample matrix, such as glucose and amino acids, with a tolerance of <0.5 Da. To minimize noise and false positives, features were required to be present in at least 75 % of the samples within a given group (i.e., 75 % of the nine samples, corresponding to 3 experimental replicates, each with triplicate extraction replicates). Features not meeting this criterion were excluded from further analysis. Additionally, an intensity threshold of at least 10^6^ counts was applied to exclude noise peaks.

For metabolite annotation, an annotation list was created that included metabolites of bacterial origin. The annotation list was based on HMDB 5.0 (Human Metabolome Database) fecal metabolites (Wishart et al., 2022) and MetaCyc (Caspi et al., 2020).

Metabolites were annotated if the mass error was less than 2 ppm and the mSigma (isotope error) was less than 500. For annotation, datasets from both positive and negative ion modes were merged for each of the two mass ranges. Potential ions considered included [M+H]^+^, [M+Na]^+^, and [M+K]^+^ in positive mode, and [M-H]^-^ and [M+Cl]^-^ in negative mode. The data tables were then exported to R (2024) for further preprocessing. Sample replicates were combined by calculating the median intensity if the molecular formula was present in at least two of the three replicates. If this condition was not met, the molecular formula was excluded to avoid noise contamination. If a molecular formula was detected by both methods (in two different mass ranges), the dataset with the lower number of non-detects (NA) was prioritized. If both datasets had the same number of non-detects, the dataset with the highest median intensity across all samples was selected. Subsequently, the intensities of metabolites detected in both hydrophilic and lipophilic phase datasets were summed, as the separation into different result datasets was performed during sample preparation (Brix et al., 2024). NA values were replaced either with 999,999 counts (the limit of detection (10^6^) minus one) if 50 % or more values were missing or by the k-nearest neighbor method if more than 50 % of the values were detected.

### 2.7 Statistics metabolomics analyses

The statistical software R (2024) was used to evaluate the data. For each metabolite separately, the data analysis started with the definition of an appropriate statistical model based on generalized least squares (Box et al., 1994). The model included sample and time, as well as their interaction term. The correlations of the measurement values due to the different time points were taken into account. The residuals were assumed to be approximately normally distributed and to be homoscedastic. These assumptions are based on graphical residual analysis. Based on this model, multiple contrast tests (Bretz et al., 2011; Hothorn et al., 2008) were performed to compare the different levels of sample and time, respectively. The number of metabolites was also taken into account in the calculation of the p-values. The PCA (principal component analysis) score plots were generated in MetaboAnalyst 5.0 (Xia et al., 2009), with settings according to Jensen-Kroll et al. (2025).

### 2.8 Pathway enrichment analyses, metabolite classification, and grouping

Only significant metabolites were included in the pathway enrichment analyses and metabolite grouping. The pathway enrichment analyses were performed using KEGG Mapper (Kanehisa et al., 2022; Kanehisa and Sato, 2020). Grouping was based on the assignment of metabolites to substance classes according to the HMDB taxonomy (Wishart et al., 2022). Groups were determined at the molecular formula level in order to avoid an overrepresentation of isobars. Therefore, each substance class was counted at most once per molecular formula. Substance classes to which only one metabolite was assigned were summarized under “Other”.

## 3 Results and discussion

The aim of this study was to analyze the metabolomic changes in bacterial communities in response to the plant compounds rutin and genistein, focusing on differences related to BMI categories. The experiments were conducted using pooled fecal samples from different individuals (**see 2.1**). The purpose of pooling is to mask the individual microbiomes and allow a greater focus on the microbial community associated with dysbiosis.

### 3.1 Metabolomic changes in the bacterial communities

**Fig. 1:**
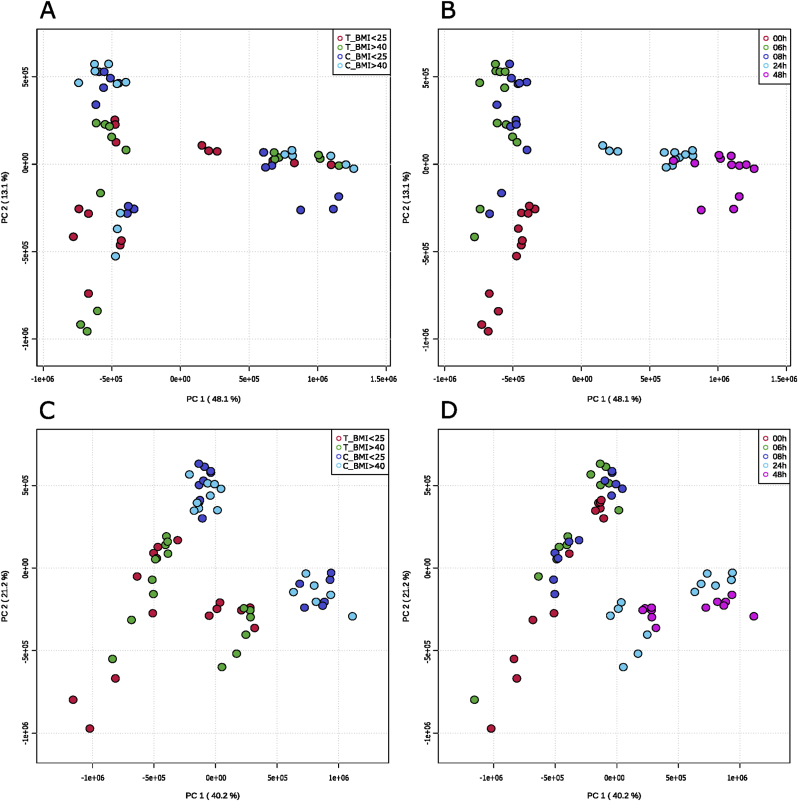
**PCA score plots of the metabolomics analyses**. PCAs performed with MetaboAnalyst 5.0 using the obtained merged MS data with Pareto scaling. All features with a matched chemical formula were included. Both time-dependent and treatment-dependent metabolomic changes were detected. **A/B** Rutin treatment, **C/D** Genistein treatment, **A/C** Color-coded by sample (Treatment BMI <25/BMI >40, Control BMI <25/BMI >40), **B/D** Color-coded by time (0–48h), each dot represents one experimental replicate, the corresponding extraction replicates were summarized.

Both incubation time- and plant compound-related metabolomic changes were observed. For rutin, primarily time-dependent changes were evident in the PCA score plots (Fig. 1A/B), but there were no clear differences between control samples and rutin-treated samples. Three distinct clusters could be identified, one at 0h, one at 6 and 8h, and another at 24 and 48h (Fig. 1B). Only at 6 and 8h incubation time could a slight separation of controls and rutin-treated samples be observed (Fig. 1B).

In contrast, a clear differentiation between genistein-treated samples and controls was observed (Fig. 1C). Depending on the incubation time, two distinct clusters could be identified, i.e., at 0, 6, and 8 h, as well as at 24 and 48 h, respectively (Fig. 1D).

**Fig. 2:**
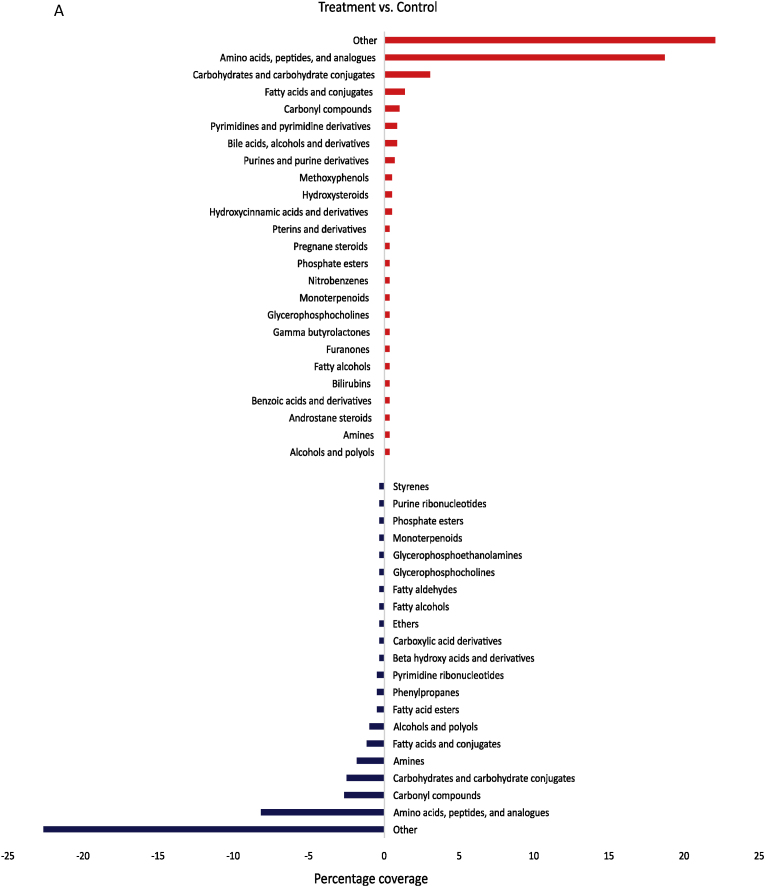

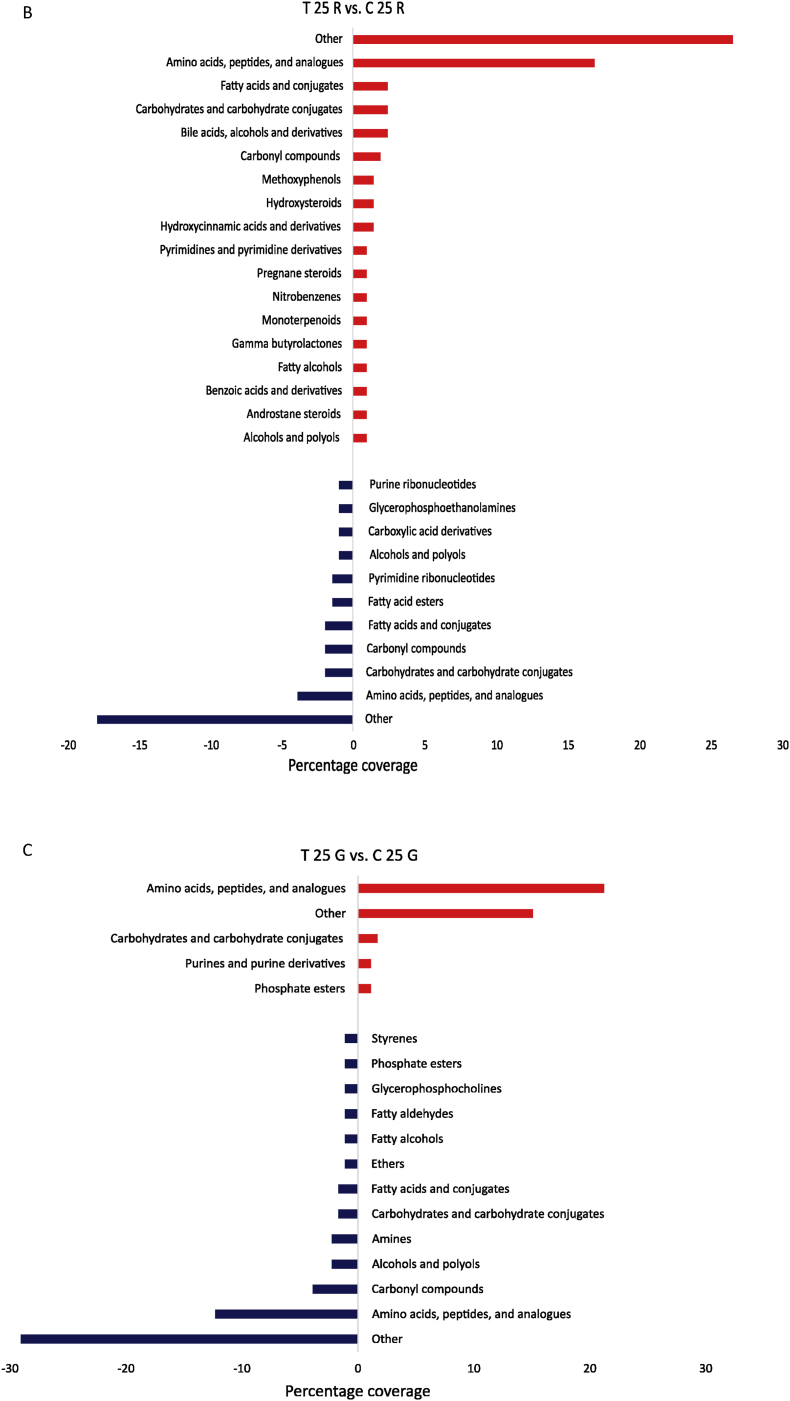

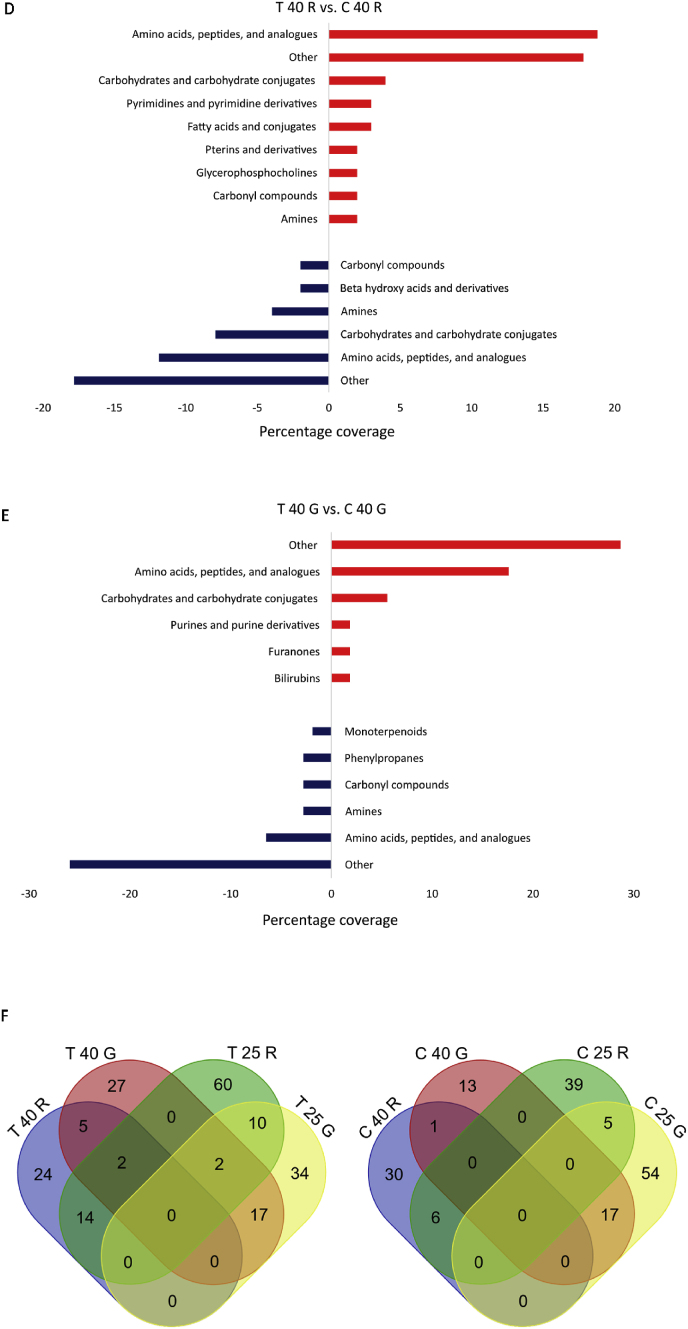
**Changes in the metabolome at the level of metabolites and corresponding substance classes**. **A** Overview of the main substance classes affected by genistein (G) and rutin (R) treatments. Substance classes increased by the polyphenols are shown in red, substance classes decreased by the polyphenols are shown in blue. Higher in the treatments (T) also means lower in the controls (C) and vice versa. Substance classes with only one metabolite were grouped under “Other”. **B** Substance classes of the BMI <25 comparison with rutin, **C** Substance classes of the BMI <25 comparison with genistein, **D** Substance classes of the BMI >40 comparisons with rutin and **E** Substance classes of the BMI >40 comparison with genistein. The shown substance classes are based on HMDB nomenclature. **F** Venn diagrams calculated on molecular formulas, treatments (left), controls (right). Only significantly regulated metabolites were included.

In the following, only significant changes in metabolite intensities were considered. At the molecular formula level, a total of 361 compounds were significantly regulated in their response by rutin and genistein (p-value <0.05) compared to the corresponding controls. The metabolites identified in this study were categorized into 35 different substance classes based on the HMDB chemical taxonomy. The category “Other” summarizes all compounds that were present as a single metabolite in other substance classes except those listed in Fig. 2. A global overview (Fig. 2A) of all treatments and corresponding controls showed that certain substance classes were both positively and negatively influenced by the plant compounds. This was evidenced by higher or lower contents in the samples treated with plant compounds compared to the control without plant compounds. These included alcohols and polyols, amines, amino acids, peptides, and analogues, carbohydrates and carbohydrate conjugates, carbonyl compounds, fatty acids and conjugates, fatty alcohols, glycerophosphocholines, monoterpenoids, and phosphate esters. Positively correlated metabolites corresponded to bilirubins, hydroxysteroids, hydroxycinnamic acids and derivatives, nitrobenzenes, and pregnane steroids. Negatively correlated metabolites, on the other hand, corresponded to carboxylic acids and derivatives, ethers, fatty acid esters, fatty aldehydes, purine ribonucleotides, pyrimidine ribonucleotides, and styrenes. The substance classes most affected on both sides were amino acids, peptides, and derivatives, carbohydrates and carbohydrate conjugates, carbonyl compounds, and fatty acids and conjugates. Amino acids, peptides, and derivatives tended to be most affected in the plant compound-treated samples, and the amounts of carbonyl compounds decreased. Moreover, substance classes such as pyrimidines and pyrimidine derivatives, bile acids, alcohols and derivatives, and purines and purine derivatives were found to be of minor importance in the controls, while amines and alcohols and polyols were of minor importance in the treatments.

When the two different BMI pools (Fig. 2B–E) were compared to their respective controls, it was evident that more substance classes were affected in the BMI <25 pools (Fig. 2B/C) compared to the BMI >40 pools (Fig. 2D/E). Furthermore, a higher number of metabolites was regulated in the BMI <25 pools. I.e., the pools with BMI <25 show a higher diversity in the response to genistein and rutin than the pools with BMI >40, as demonstrated in the Venn diagrams (Fig. 2F). For example, a total of 45 significantly regulated metabolites accumulated differently for rutin-treated feces in the BMI >40 pools compared to 88 metabolites in the BMI <25 pools. The higher diversity of regulated metabolites in BMI <25 vs. BMI >40 corresponds to a higher α-diversity of the microbiome as determined by 16S rRNA gene metagenomics (Jensen-Kroll et al., 2025). This suggests that a higher diversity in the response may be associated with a higher diversity of the microbial composition. It is known that there is a decrease in gut microbial diversity in the context of obesity, as was first described by Turnbaugh et al. (2009). Nicholson et al. (2012) postulated that the state or condition of the intestinal microbiome is reflected in human biofluids such as serum and can be determined by metabolomics.

### 3.2 Changes in amino acids, peptides, amines, and corresponding pathways

Amino acids, peptides, and analogues were the class of metabolites most affected by the plant compounds in both BMI pools (Fig. 2A). Approximately one third of all significantly regulated metabolites belonged to this category. 80 out of 120 metabolites were significantly increased, while 40 were decreased by the genistein and rutin treatments, and it was particularly striking that half of these were dipeptides (53 in treatments/14 in controls).

It was also noteworthy that specifically the dipeptides containing either glutamine or tryptophan were significantly increased in the plant compound-treated samples, glutamine in both BMI pools, and tryptophan-containing dipeptides only in the BMI <25 pools. In general, glutamine was the predominant amino acid in the dipeptides of the plant compound-treated samples, while aspartic acid was predominant in the corresponding controls in both BMI pools.

Dipeptides are formed by the degradation of proteins. Many bacteria have extracellular proteases that cleave proteins into polypeptides, oligopeptides, or individual amino acids. The resulting amino acids, as well as the di- and tripeptides, can be taken up by the bacterial cell through various transport systems. Di- and tri-peptides are subsequently cleaved into amino acids by intracellular peptidases, which are then used for energy metabolism or for the cell's own protein biosynthesis (Munk and Dersch, 2009). Dipeptides in general are also associated with signaling functions and antioxidant capacity (Verbeke et al., 2017; Covasa et al., 2019; Ozawa et al., 2022; Kaushik et al., 2022). The observed increased occurrence of dipeptides in the analyzed cell supernatants could theoretically have different causes. It could be due to accumulation, due to inhibited transport or cleavage of the dipeptides, or the result of increased formation. Yoda et al. (2004) and Nakayama et al. (2013) used epigallocatechin gallate (EGCG) and demonstrated that polyphenols can interact with the porins located in the outer membrane of Gram-negative bacteria such as *E. coli*. They could block these and inhibit the transport of biomolecules, such as amino acids or dipeptides (Makarewicz et al., 2021). This could theoretically explain an increase in such molecules also in our study in the presence of the plant compounds. Furthermore, as reviewed by Alvarez et al. (2010), flavonoids may additionally modulate and influence the transport of biomolecules via ABC transporters. Singh et al. (2020) showed that quercetin and coumarin inhibited dipeptidyl-peptidase IV, and thus the inhibition of cleavage cannot be excluded as a possible further reason for the accumulation of the dipeptides in this study. In this context, an explanation may be that the previously observed decrease in proteolytically active *Clostridiaceae* (Jensen-Kroll et al., 2025) may have affected the observed dipeptide accumulation. As previously described, glutamine was the most abundant amino acid among the identified dipeptides in both BMI pools treated with plant compounds. However, considering the general composition of proteins, glutamine is not the most abundant amino acid (Bruice, 2007), so this finding is particularly noticeable. Glutamine has been described as one of the most versatile amino acids, as all other amino acids, as well as nucleic acids, glutathione, amino sugars, hormones such as GABA, and co-enzymes, can be synthesized from glutamine via glutamate (Cruzat et al., 2018). Glutamine is also associated with a beneficial effect in the context of obesity and type 2 diabetes (T2D), as it promotes the release of the intestinal hormone glucagon-like peptide 1 (GLP-1). This inhibits the release of glucagon and increases the glucose sensitivity of the beta cells in the pancreas, which in turn stimulates the release of insulin and lowers blood glucose levels (Greenfield et al., 2009). GLP-1 furthermore inhibits the apoptosis of beta cells and promotes the proliferation and differentiation of these insulin-producing cells (Reed et al., 2020). Therefore, the increase in glutamine in dipeptides as a result of treatment with plant compounds may be viewed as a beneficial outcome. Tryptophan, which was also found to be present in a higher abundance in dipeptides, can, on the other hand, be viewed more critically in the context of obesity, as it is associated with an increased BMI and adipose tissue mass (Lischka et al., 2022).

The tryptophan metabolism holds a unique role among the amino acids concerning obesity, as it has been described as dysregulated in this context. Tryptophan is primarily degraded through three main pathways: approximately 90 % via the kynurenine pathway, with lesser amounts through the serotonin and indole pathways (Hou et al., 2023). Research by Cussotto et al. (2020) revealed that the kynurenine to tryptophan ratio is significantly higher in obese individuals compared to non-obese individuals. The BMI <25 samples treated with genistein showed significantly lower levels of kynurenic acid compared to the controls not treated with genistein, which could be beneficial as it positively influences the aforementioned ratio. However, in samples with a BMI >40, a significantly lower content of indole-3-propionic acid was observed. Additionally, significantly higher levels of 5-hydroxytryptophan, indole-3-acetic acid, and indole-3-acrylic acid were detected in the rutin-treated samples with BMI <25, indicating that polyphenols may stimulate alternative degradation pathways. Indole-acrylic acid is known for its anti-inflammatory properties (Wlodarska et al., 2017). Furthermore, polyphenols seem to affect tryptophan degradation primarily in samples with BMI <25. The fact that the tryptophan metabolism is influenced by polyphenols was also shown in an intervention study by Ulaszewska et al. (2020). They also demonstrated that tyrosine metabolism is influenced by polyphenols. However, this may also be due to an overlap of metabolites, resulting from the degradation of polyphenols. Examples are hydroxyphenylacetic acid, hydroxyphenylpropionic acid, and p-cresol (Jensen-Kroll et al., 2025).

As already shown for the substance classes (Fig. 2), it was evident that the BMI <25 pools showed a higher diversity in the response to the plant compounds. All amino acids were more abundant in the BMI <25 pools, except for threonine, serine, and arginine.

A total of ten free amino acids were detected; five in the treatments and five in the controls. Significantly higher levels of glycine were detected in the rutin treatments in both BMI pools. Threonine levels occurred in higher amounts only in the BMI <25 pools, both with rutin and genistein. The corresponding controls showed higher levels of histidine. Proline and valine were lower in the genistein treatments (both BMI pools). The higher glycine levels are also of particular interest in the context of obesity. Glycine is a key player in the human detoxification system and has been described as dysregulated in this context (Tan et al., 2024). All significantly regulated dipeptides and amino acids, as well as all other annotated metabolites, are shown in the supplementary tables.

Additional pathway enrichment analysis based on KEGG mapping resulted in 15 active pathways related to amino acid metabolism. The pathways with the significantly regulated metabolites and the plant compound treatment-based regulations are shown in Table 1. Based on the total number of hits over all 361 significantly regulated metabolites, lysine degradation, histidine metabolism, and arginine and proline metabolism were more affected by the plant compounds. Considering only the metabolites that are specific to one pathway and not those that are part of multiple pathways, it was observed that 10 out of 15 both significantly upregulated and significantly downregulated metabolites occurred within one pathway, but in some cases in different modules of the pathway. For example, this was the case for the lysine degradation pathway. The significantly regulated metabolites of arginine biosynthesis, phenylalanine, tyrosine and tryptophan biosynthesis, and tyrosine metabolism detected were increased in response to the treatments with the plant compounds. The metabolites of the phenylalanine metabolism and lysine biosynthesis were decreased by plant compound treatments. This also applied to the most metabolites of the alanine, aspartate, and glutamate metabolism, as well as the histidine metabolism.

**Table 1:**
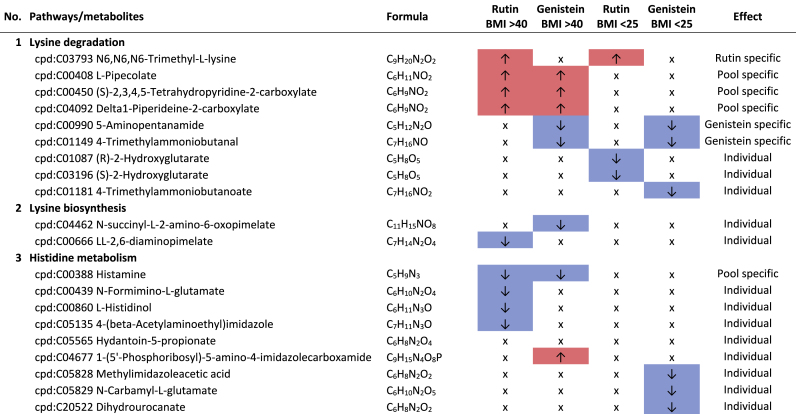

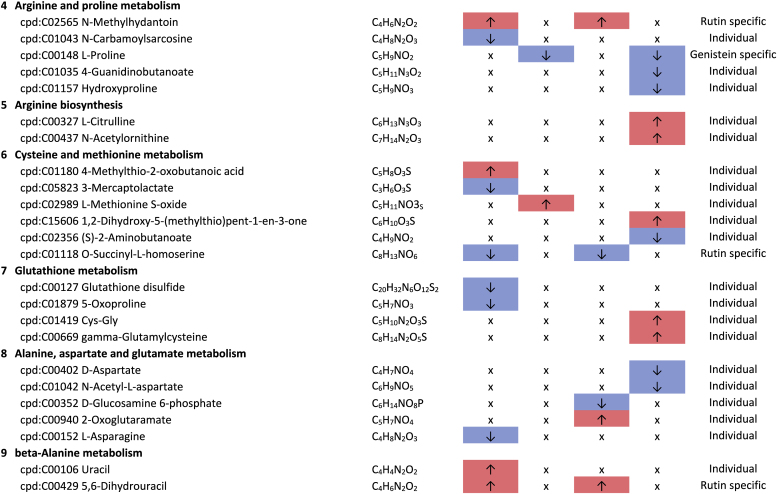

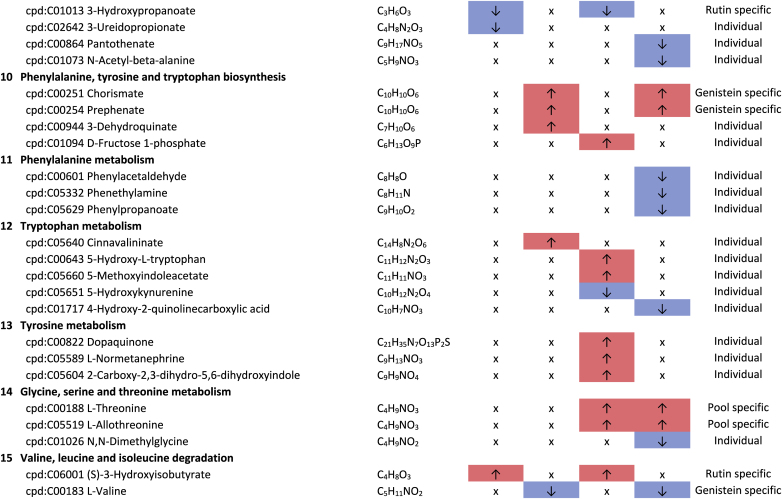

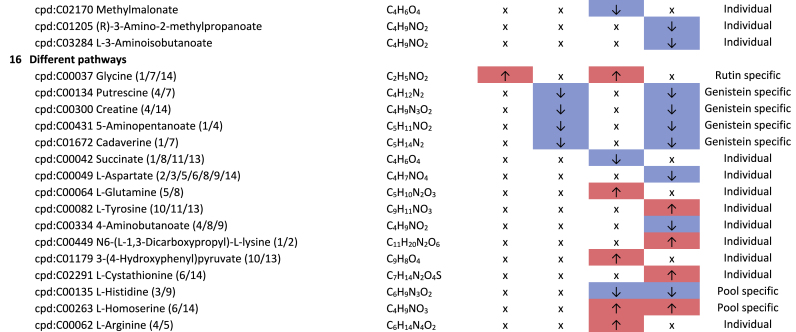
**Results KEGG-mapping of significantly regulated metabolites to pathways of the amino acid metabolism** with KEGG-IDs, molecular formulas, and effects, separated by treatments, pools, and pathways; ↑ sig. higher abundance in the treated samples in comparison to the corresponding control without plant compound, ↓ sig. lower abundance in comparison to the corresponding control without plant compound; metabolites that are part of several pathways are listed under “Different pathways”.

In addition to the amines associated with the amino acid pathways, histamine, cadaverine, putrescine, 4-aminobutanoate (GABA), and histidinol (Table 1), six further amines were detected. A treatment-based, significant increase of ethanolamine and porphobilinogen and a decrease of stearoylethanolamide occurred in the BMI >40 pool in dependence on rutin. In the corresponding controls of the genistein-treated sample of the BMI <25 pools, higher intensities of propylamine, trimethylamine, and phenylethylamine were observed.

Biogenic amines can result from the enzymatic activity of decarboxylases and aminases from amino acids, aldehydes, and other nitrogen-containing compounds (Tsafack and Tsopmo, 2022). Histidinol and histamine are derived from the amino acid histidine, cadaverine from lysine, and putrescine from arginine. The associated metabolic pathways are also the pathways most affected by the plant compounds. Biogenic amines can have both positive and negative effects on human health. For example, they can act as neurotransmitters, be involved in pro-inflammatory signaling, be part of co-enzymes, and be precursors of other hormones and other biogenic amines (Erdag et al., 2019; Saha et al., 2024; Ruiz-Capillas and Herranz, 2019). In large amounts, they can also have a toxic effect (Silla Santos, 1996).

Of particular health relevance in this study may be the reduction of histamine, histidinol, and trimethylamine (TMA), and the increased formation of ethanolamine in association with rutin and genistein. The TMA produced in the intestine by associated bacteria can be oxidized to trimethylamine N-oxide (TMAO) in the liver after absorption. Elevated levels of TMAO are associated with an increased risk of cardiovascular diseases, particularly heart attack and stroke (Tang et al., 2021; Zhang and Yao, 2023). The lower levels of TMA in association with genistein in the BMI <25 pool are therefore considered positive.

The occurrence of lower levels of histamine in both BMI >40 pools and histidinol in the BMI >40 pools with rutin is also considered to be a positive effect. Histidinol is a precursor of histamine, and histamine acts in the human body as a tissue hormone and neurotransmitter with a central role in allergic reactions and inflammation (Branco et al., 2018; Hirasawa, 2019). This is also interesting with regard to the fact that obesity is associated with a low-grade inflammation (Khanna et al., 2022). The fact that polyphenols can have an inhibitory effect on the formation of biogenic amines has also been described by Tsafack and Tsopmo (2022). On the other hand, a higher level of ethanolamine in the BMI >40 pool treated with rutin is considered to be detrimental in the context of obesity, as it is known to have a negative effect on the intestinal barrier, which is already compromised in the case of obesity (Mishra et al., 2023).

### 3.3 Changes in carbohydrates, carbohydrate derivatives, and corresponding pathways

The second most common class of metabolites affected by the plant compounds were carbohydrates and carbohydrate derivatives. Due to their isobarity, some of the carbohydrates could not be distinguished, that only the superordinate substance class is mentioned. The pathway enrichment analyses in Table 2 indicate which metabolites are potentially involved. In particular, higher levels of various monosaccharides and disaccharides were observed. In 3 out of 4 pools treated with plant compounds (rutin BMI >40 and BMI <25, and genistein BMI <25), significantly higher levels of hexoses (C_6_H_12_O_6_) were detected compared to the respective controls without plant compounds. Furthermore, a significantly higher content of disaccharides with the molecular formula C_12_H_22_O_11_ was found in the BMI >40 pools treated with rutin or genistein. Depending on genistein, an additional disaccharide with the molecular formula C_12_H_20_O_11_ was detected in the BMI >40 pool, and in both BMI <25 and BMI >40 pools, genistein-dependent disaccharides with the molecular formula C_10_H_18_O_9_ were also observed. Moreover, higher levels of pentoses (C_5_H_10_O_5_) were detected in the BMI <25 pools depending on rutin. It is known that polyphenols can affect the phosphotransferase system (PTS) and thus sugar transport (Makarewicz et al., 2021; Firrman et al., 2018), suggesting an accumulation by inhibition of sugar transport.

**Table 2:**
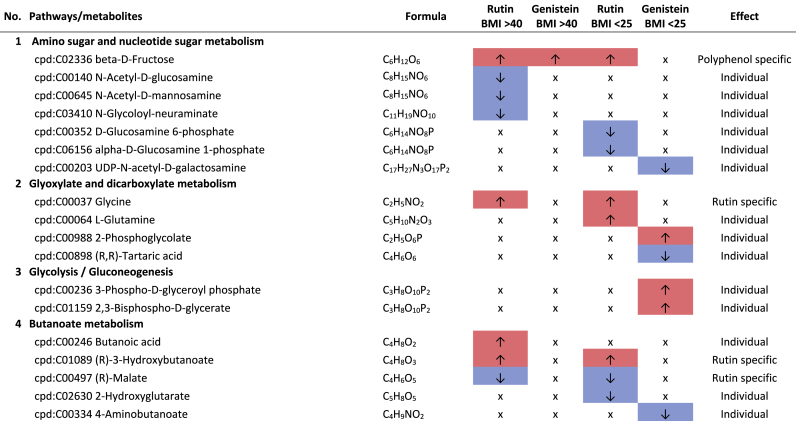

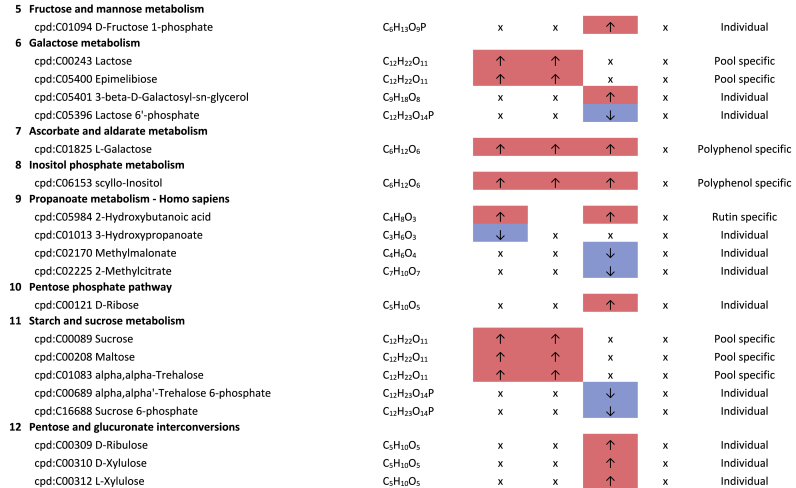

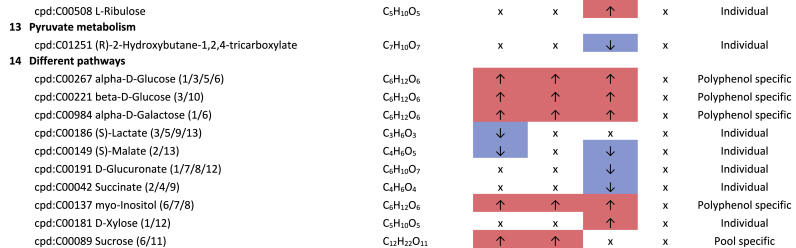
**Results KEGG-mapping of significantly regulated metabolites to pathways of the carbohydrate metabolism** with KEGG-IDs, molecular formulas, and effects, separated by treatments, pools, and pathways; ↑ sig. higher abundance in the treated samples in comparison to the corresponding control without plant compound, ↓ sig. lower abundance in comparison to the corresponding control without plant compound; metabolites that are part of several pathways are listed under “Different pathways”.

Depending on the polyphenols rutin and genistein, lower levels of various amino sugars and derivatives were found compared to the controls without plant compounds. Specifically, in relation to rutin, significantly lower levels of N-glycoloylneuraminate and N-acetyl-D-glucosamine/N-acetyl-D-mannosamine were detected in the BMI >40 pools when compared to the controls. Additionally, lower levels of D-glucosamine-phosphate and D-glucuronate were observed in the BMI <25 pools, along with lower levels of N-acetyl-neuraminic acid in both BMI pools dependent on rutin. Also, genistein-dependent, lower levels of lactosamine were detected in the BMI <25 pools.

The basic structure of the bacterial cell wall consists of peptidoglycans, i.e., the murein. The murein layer is composed of strands of β1-4 glycosidically linked N-acetylglucosamine with N-acetylmuramic acid. Each N-acetylmuramic acid is connected to another N-acetylmuramic acid molecule of an adjacent strand via an oligopeptide chain. Attached to each N-acetylmuramic acid molecule are tetrapeptides that can be cross-linked by additional amino acids or directly through an interpeptide bridge. Within the tetrapeptides, both L- and D-amino acids occur, in particular D/L-alanine and D-glutamine in both Gram-negative and Gram-positive bacteria, whereas most Gram-negative bacteria, e.g., *Bacteroides*, contain diaminopimelic acid, and Gram-positive bacteria use mainly lysine instead (Triassi et al., 2014). In the present experiments, the levels of diaminopimelic acid were also significantly lower in the rutin-treated samples of the BMI >40 pools compared to the corresponding controls without plant compounds.

N-acetylneuraminic acid and glucosamine-phosphate can be precursor molecules for N-acetyl-D-glucosamine. Lower levels associated with polyphenols suggest that polyphenols inhibit cell wall synthesis. The different effects of polyphenols on cell wall synthesis were also described by Makarewicz et al. (2021). Since diaminopimelic is primarily associated with Gram-negative bacteria, it can be assumed from the data that Gram-negative bacteria are particularly affected. This can be considered beneficial if Gram-negative bacteria such as (opportunistic) pathogens (e.g. genera of gut-associated *Enterobacteriaceae*) would be affected.

Furthermore, significantly lower levels of lactate were detected in the BMI >40 pools treated with rutin compared to the untreated samples without rutin. Lactate is the end product of homofermentative or heterofermentative lactic acid fermentation and is produced in the intestine by various bacteria, including lactic acid bacteria and bifidobacteria (Sauer et al., 2017). In the samples treated with rutin, significantly lower abundances of *Bifidobacteriaceae* in both BMI pools and lower levels of *Lactobacillaceae* in the BMI >40 pool were detected (Jensen-Kroll et al., 2025). In the context of obesity, lower lactate levels should be considered positive, as lactate has been implicated as a key player in obesity-induced inflammation and systemic insulin resistance (Lin et al., 2022). However, the overall reduction in probiotic bacteria should be considered unfavorable.

### 3.4 Changes in the nucleotide metabolism, including pyrimidines and purines

The metabolism of pyrimidines and purines shows both up- and down-regulated metabolites affected by genistein and rutin (Table 3). In terms of the number of regulated metabolites, pyrimidine metabolism appears to be more affected by these plant compounds compared to purine metabolism, especially by rutin. Metabolites involved in both the de novo synthesis and degradation were determined. In both BMI pools, rutin-specific lower levels of inosine and higher levels of CTP and 5,6-dihydrouracil were detected when compared to the corresponding controls without plant compounds. In addition, genistein-specific higher levels of urate, xanthine, and (R)-5,6-dihydrothymine were observed in both pools. Polyphenols can have both growth inhibitory and growth promoting effects on bacteria (Makarewicz et al., 2021; Lippolis et al., 2023), as shown in previous experiments for various genera and families (Jensen-Kroll et al., 2025). It is suggested that these results may be due to this cause, but this should be investigated further.

**Table 3:**
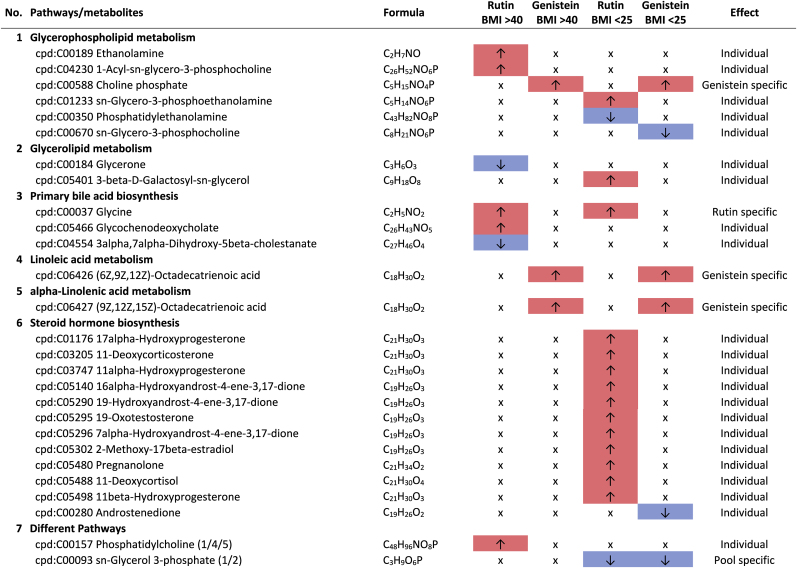
**Results KEGG-mapping of significantly regulated metabolites to pathways of the lipid metabolism** with KEGG-IDs, molecular formulas, and effects, separated by treatments, pools, and pathways; ↑ sig. higher abundance in the treated samples in comparison to the corresponding control without plant compound, ↓ sig. lower abundance in comparison to the corresponding control without plant compound; metabolites that are part of several pathways are listed under “Different pathways”.

### 3.5 Changes of fatty acids, conjugates, and the lipid metabolism

The pathway alignment showed hits to six pathways of the lipid metabolism (Table 4), indicating that lipid metabolism is affected by the phytochemicals. It was particularly noticeable that rutin led to significantly higher concentrations of the SCFA butyrate in the BMI >40 pools and to significantly higher concentrations of hydroxybutyric acid in both BMI pools compared to the corresponding control without rutin. Both polyphenols, rutin and genistein, led to significantly lower concentrations of the MCFA (medium chain fatty acid) capronate in the BMI <25 pools, and rutin-dependently also to lower levels of the SCFA valerate in the same pool. The precursor amino acid valine occurred with significantly lower intensities in the genistein-treated samples of both BMI pools.

**Table 4:**
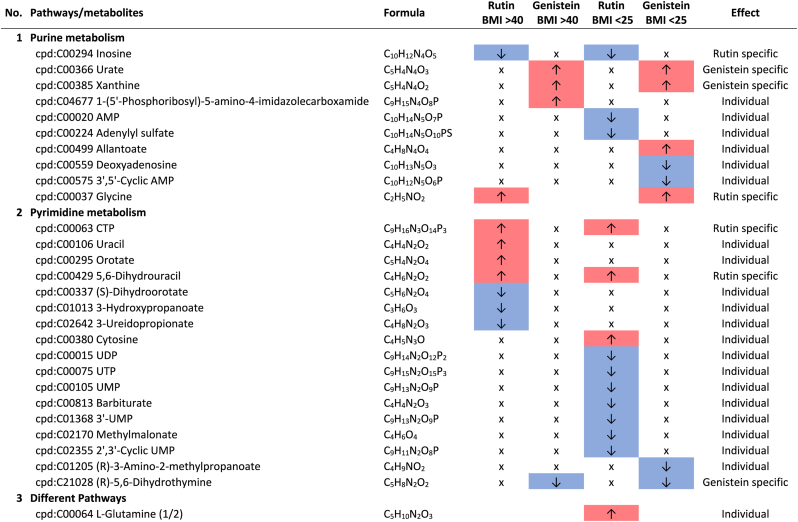
**Results KEGG-mapping of significantly regulated metabolites to pathways of the nucleotide metabolism** with KEGG-IDs, molecular formulas, and effects, separated by treatments, pools, and pathways; ↑ sig. higher abundance in the treated samples in comparison to the corresponding control without plant compound, ↓ sig. lower abundance in comparison to the corresponding control without plant compound; metabolites that are part of several pathways are listed under “Different pathways”.

There are a number of possible explanations for the increase in butyrate in the BMI >40 samples treated with rutin. Along with acetate and propionate, butyrate is a major fermentation product in the bacterial catabolism of indigestible carbohydrates. However, butyrate can also be produced from other hexoses, from the fermentation of amino acids, and via cross-feeding interactions from acetate and lactate (Louis and Flint, 2017), which could explain the reduced lactate levels in the rutin-treated BMI >40 pools. It is also possible that butyrate is the result of the degradation of quercetin, as was shown by Pakar et al. (Parkar et al., 2013). In this context, butyrate could be formed from the glycone of rutin or from the aglycone quercetin. Butyrate serves as a main energy source for colonocytes and plays a key role in maintaining colonic health and integrity (Hamer et al., 2008). However, in the context of obesity, butyrate is controversially discussed. Several studies, mostly in animals, have shown that butyrate supplementation has beneficial effects on adipose tissue metabolism, energy metabolism, and inflammation at both the systemic and tissue levels, as well as insulin sensitivity and body weight regulation (van Deuren et al., 2022; Coppola et al., 2021). Other human studies showed that obese individuals had higher fecal butyrate concentrations than non-obese individuals on similar diets and that this was influenced by weight loss. This led to the consideration that butyrate may contribute to the characteristic phenotype of obesity, based on more efficient energy harvesting from dietary fiber or the use of butyrate for de novo lipid synthesis. Other considerations suggest that reduced transport and resorption of butyrate may also be the cause of its accumulation in feces (van Deuren et al., 2022). In addition, a higher content of butyrate-forming bacteria has been observed in the context of obesity, so that a higher supply may be the cause of the higher level of butyrate in the feces of obese people (Turnbaugh et al., 2006). An increase of the main butyrate-forming genus *Roseburia* in the context of rutin and genistein in the BMI >40 pools was also detected in our experiments (Jensen-Kroll et al., 2025).

The higher concentrations of hydroxybutyric acid in the rutin-treated BMI >40 pools could be considered positive. Hydroxybutyric acid is a derivative of butyrate and a precursor of the neurotransmitter 4-aminobutanoate (GABA). Lee et al. (2022) and Wang et al. (2023) showed in a high-fat diet (HFD) mouse model that GABA may have an anti-obesity effect.

The glucogenic, essential amino acid valine is one of the branched-chain amino acids, along with leucine and isoleucine. Circulating BCAAs are significantly higher in obese children and adolescents (Newgard, 2017; Zhang et al., 2023), so lower levels are therefore to be considered positive as observed for genistein in both BMI pools.

The lower levels of caproate and valerate found in the treated BMI <25 pools detected in this study could both be of microbial origin. Caproate and valerate-forming bacteria include several strains of Clostridia species (Zhu et al., 2015). Accordingly, our experiments showed that there is a significant decrease within the *Clostridiaceae* based on the polyphenol treatments (Jensen-Kroll et al., 2025). Little is known about the systemic function of capronate and valerate. In general, MCFAs are involved in energy metabolism but can also induce systemic responses by regulating the metabolite and hormone balance and thus influencing the immune response and insulin secretion (Roopashree et al., 2021).

### 3.6 Limitations of the study design

A key limitation of the study is the small sample size and the use of pooled fecal samples from seven individuals in each pool. While pooling was chosen to reduce inter-individual variability and to focus on generalizable metabolic patterns, it inherently limits the ability to capture subject-specific responses. Furthermore, the statistical replicates in this study were derived from three independent experimental replicates of the pooled samples rather than from biological replicates of individual subjects. This approach allows for technical reproducibility but does not account for biological variability across individuals, which may influence the interpretation of results. Therefore, the findings presented here should be considered exploratory and hypothesis-generating. Future studies with larger sample sizes and individual-level replicates would be essential to validate and expand upon these results, ensuring greater robustness and generalizability. Moreover, validation through a controlled dietary intervention study would be crucial to confirm the functional relevance and causality of the observed metabolic patterns.

## 4 Conclusion

The main objective of this study was to explore the metabolomic changes in bacterial communities in response to the plant compounds rutin and genistein, focusing on differences related to BMI categories. The experiments were able to show that the body mass index (BMI) had a clear effect on the metabolomic responses, with BMI <25 pools exhibiting a more diverse metabolomic response to the plant compounds compared to the BMI >40 pools. This greater metabolic response correlated with a corresponding higher microbial α-diversity in the BMI <25 pools (Jensen-Kroll et al., 2025), emphasizing the complex relationship between host health, microbial diversity, and metabolomic profiles. The study also highlights the multifaceted effects of polyphenols.

Our results showed that many metabolic pathways were affected, demonstrating that polyphenols have a modulatory effect on the microbiota and, beyond that, on host physiology. The plant compounds had a particular effect on amino acid, carbohydrate, nucleotide, and lipid metabolism, with amino acids, peptides, and analogues being the most affected. In this regard, numerous significantly regulated dipeptides and primarily lower levels of biogenic amines were observed in the samples of both BMI pools treated with rutin and genistein. Additionally, many of the metabolites that accumulated differently in this study are associated with chronic diseases. Moreover, their accumulation partly differed depending on the BMI of the feces donor group. In this context, regulations of metabolites could be observed that could potentially result in either a positive or a negative impact on health. For example, in the context of obesity, beneficial effects of rutin were lower levels of lactate (rutin BMI >40) and higher levels of butyrate (rutin BMI >40) and glycine (rutin BMI <25 and BMI >40). This is because lactate and glycine are considered to be dysregulated in the context of obesity. However, the higher levels of ethanolamine (rutin BMI >40) can be considered detrimental, as they are associated with negative effects on the intestinal barrier. In general, BMI-specific, polyphenol-specific, rutin-specific, genistein-specific and individual effects (i.e. related to a single polyphenol/specific BMI pool) were observed. This suggests the possibility that the application of polyphenols may not be equally suitable for everyone and thus may not lead to the same results or potential beneficial effects for all individuals, emphasizing the idea of personalized nutrition. Therefore, results of the explorative study would require validation with a larger sample size, biological replicates and/or an intervention study.

Overall, this research provides valuable insights into how rutin and genistein affect bacterial metabolomics, with potential implications for understanding their role in metabolic health and obesity. The methodological approach used in this study could also be applied to evaluate the effects of further plant compounds, drugs, and dietary components on the microbiota in healthy individuals or in individuals with other pathological conditions other than obesity.

## CRediT authorship contribution statement

**Julia Jensen-Kroll:** Conceptualization, Data curation, Formal analysis, Investigation, Methodology, Validation, Visualization, Writing – original draft. **Tobias Demetrowitsch:** Conceptualization, Data curation, Formal analysis, Investigation, Methodology, Supervision, Validation, Writing – review & editing. **Sabrina Sprotte:** Conceptualization, Investigation, Methodology, Supervision, Validation, Writing – review & editing. **Fynn Brix:** Data curation, Formal analysis, Investigation, Methodology, Writing – review & editing. **Alexia Beckmann:** Resources, Writing – review & editing. **Kristina Schlicht:** Writing – review & editing. **Matthias Laudes:** Funding acquisition, Project administration, Resources, Writing – review & editing. **Mario Hasler:** Formal analysis, Investigation, Methodology, Resources, Writing – review & editing. **Charles M.A.P. Franz:** Methodology, Resources, Supervision, Writing – original draft, Writing – review & editing. **Karin Schwarz:** Conceptualization, Funding acquisition, Methodology, Project administration, Resources, Supervision, Writing – original draft, Writing – review & editing.

## Data availability statement

The original contributions presented in this study are included in the article or supplementary material. For further inquiries, please contact the corresponding author.

## Declaration of generative AI in scientific writing

The authors declare that no AI was used in writing any part of the manuscript.

## Funding sources

This project was funded by the Federal Ministry of Food and Agriculture as part of the “Gut Metabotypes as Biomarkers for Nutrition and Health (BioNUGUT)” project, Grant No. 2816ERA13E.

## Declaration of competing interest

The authors declare that the research was conducted in the absence of any commercial or financial relationships that could be construed as a potential conflict of interest.

## Data Availability

Data will be made available on request.
